# McIntire Prize*Reprinted from the *Journal of Sociologic Medicine*, December, 1916.

**Published:** 1917-02

**Authors:** 


### McIntire Prize.*

Last year Dr. Charles McIntire resigned the secretaryship of the American Academy of Medicine after twenty-five years of faithful service. In appreciative commemoration the American Academy of Medicine decided to raise a fund, the income of which should be expended in accordance with Dr. McIntire's suggestions. As a consequence the Academy now announces two prize offers, the prizes to be awarded at the annual meetings for 1918 and 1921, respectively.

The subject for 1918 is "The Principles Governing the Physician's Compensation in the Various Forms of Social Insurance." The members of the committee to decide the relative value of the essays awarding this prize are—Dr. John L. Heffron, Dean of the College of Medicine, Syracuse University; Dr. Reuben Peterson, Professor of Obstetrics and Diseases of Women, University of Michigan, and Dr. John Staige Davis, Professor of Pediatrics and Practice of Medicine, University of Virginia.

The subject for 1921 is "What Effect Has Child Labor on the Growth of the Body?" The members of the committee to award this prize are—Dr. Thomas S. Arbuthnot, Dean of the Medical School of the University of Pittsburg; Dr. Winfield Scott Hall, Professor of Physiology, Northwestern University, and Dr. James C. Wilson, Emeritus Professor, Practice of Medicine and of Clinical Medicine, Jefferson Medical College.

The conditions of the contests are:

1. The essays are to be typewritten and in English, and the contests are to be open to everyone.

2. Essays must contain not less than 5000 more than 20,000 words, exclusive of tables. They must be original and not previously published.

3. Essays must not be signed with the true name of the writer, but are to be identified by a nom de plume or distinctive device. All essays are to reach the Secretary of the Academy on or before January 1st of the years for which the prizes are offered and are to be accompanied by a sealed envelope marked on the outside with the fictitious name or device assumed by the writer and to contain his true name inside.

4. Each competititor must furnish four copies of his competitive essay.

---

*Reprinted from the *Journal of Sociologic Medicine*, December, 1916.

5. The envelope containing the name of the author of the winning essay will be opened by Dr. McIntire, or in his absence by the presiding officer at the annual meeting and the name of the successful contestant announced by him.

6. The prize in 1918 for the best essay submitted according to these conditions will be $100; that of 1921 will be $250.

7. In case there are several essays of especial merit, after awarding the prize to the best, special mention of the others will be made and both the prize essay and those receiving special mention are to become at once the property of the Academy, probably to be published in the *Journal of Sociologic Medicine.* Essays not receiving a prize or special mention will be returned to the authors on application.

8. The American Academy of Medicine reserves the right to decline to give the prize if none of the essays are of sufficient value.

The present officers of the American Academy of Medicine are: George A. Hare, M. D., Fresno, California, President; J. E. Tuckerman, M. D., Cleveland, President-elect; Charles McIntire, M. D., Easton, Pa., Treasurer, and Thomas Wray Grayson, M. D., 1101 Westinghouse Building, Pittsburg, Pa., Secretary.

---

Dr. J. Frank Clark, formerly of Iowa Park, has opened a modern twenty-bed hospital at Alpine, Texas.

